# What can be achieved in the aeromedical environment in 2012?

**DOI:** 10.1186/1757-7241-21-S1-A4

**Published:** 2013-05-28

**Authors:** P Avery, DJ Lockey

**Affiliations:** 1 Brighton and Sussex Medical School, Brighton, UK; 2 North Bristol NHS Trust & London’s Air Ambulance, UK

## 

The first day of this year’s London Trauma Conference explored the functions and scope of Air Ambulances. The meeting was hosted by the Norwegian Air Ambulance and had speakers from Scandinavia, UK, US, Austria and Switzerland. Key subject areas included system development, patient safety and optimal management of specific clinical problems in the air ambulance environment.

## Systems

The first speaker was Colonel Peter Kovats, United States Air Force, Chief of Aerospace Medicine at RAF Lakenheath UK. He spoke on Aeromedical Evacuation – ‘*from point of wounding all the way home’*. Colonel Kovats gave an extensive review of intra/inter-theatre air-evacuation and discussed 7656 patients injured in Operation Enduring Freedom in 2010. 15% were military coalition casualties and over 90% survived. Impressive survival rates of up to 95% with massive transfusion were presented. He suggested that key areas for improved care include cranio-facial reconstruction, burn repair with cell engineered skin, prevention of compartment syndromes, healing without scaring with cell spraying, limb and digit salvage, and muscle regeneration. Cornel Kovats stated that the system has developed to deliver the same level of care in transport as in hospital. This was a persistent theme in presentations at the conference – the concept that patients condition rather than location should determine the level of care provided.

Mr Graham Chalk, Lead Paramedic London’s Air Ambulance spoke knowledgably on *dispatch of air ambulance services in trauma*. This topic was identified at a recent consensus meeting as one of the top 5 research priorities in pre-hospital practice. Mr Chalk’s comprehensive review of the literature outlined current methods used for dispatch. The traditional dispatch using mechanism of injury was demonstrated to be a poor discriminator for HEMS dispatching. Critically, multiple studies demonstrate physiological criteria were most accurate for dispatch, but this may be critically influenced by the reliability and experience of on-scene personnel. The current approach of London’s Air Ambulance utilises clinical interrogation dispatch in addition to seven immediate dispatch criteria. The data presented suggested interrogation dispatch is as accurate as on-scene ambulance service requests but leads to dispatch on average 13 minutes quicker. Immediate dispatch was 10% less accurate, but on average 4 minutes quicker. It was concluded that criteria can be created which have sensitivity and specificity but should be used in a flexible manner by clinically experienced individuals in the clinical dispatch centre.

The controversial question of pre-hospital trauma triage: *only a matter of flowcharts or does provider competence matter?* was addressed by Dr Marius Rehn a Senior Researcher from the Norwegian Air Ambulance. Barriers to field trauma triage were summarised, with some thoughts on how to overcome them. Some triage generalisations were of particular interest. Under-triaged patients tended to be older, whereas over-triaged patients are younger and commonly involved in road traffic collisions. It was concluded that a shift of focus is required to improve triage and point of care diagnostics may be helpful. Evidence collected using the Utstein trauma template for uniform reporting of data following major trauma may be useful in measuring the effectiveness and features of good triage.

Dr Nils Petter Oveland, also from the Norwegian Air Ambulance, talked passionately about ultrasound applications in HEMS, provocatively asking *is it time to dismiss the stethoscope?* By describing ultrasound as a way of identifying pneumothorax, haemothorax, fracture, tendon rupture, optic nerve diameter for ICP, confirming tracheal tube placement, as well as an extended FAST protocol, he attempted to convince the audience that ultrasound is an essential component of the air ambulance tool kit. Focused assessed transthoracic echo ‘FATE’ was suggested for scanning shocked patients as correct pre-hospital management is dependant on successful diagnosis. Dr Overland concluded by highlighting procedural applications in addition to diagnostic benefits. The talk demonstrated that ultrasound is feasible in the pre-hospital environment and has many potential applications. Exactly which examinations change management and justify delay on scene is a key question for the future. Professor Hans Morten Lossius, director of research and development at Norwegian Air Ambulance, presented the case for stroke thrombolysis in pre-hospital care. German ambulances dedicated to stroke with mobile head CT scanners, neurologists, and neuroradiologists have demonstrated that a large reduction in time from symptom onset to thrombolysis is possible. By achieving an average of 72minutes to thrombolysis, the patient is treated within the 2 hour period of maximum effect. Professor Lossius presented a clinical and economic case for a helicopter stroke service that could be utilised for a population of around 1 million to deliver early stroke thrombolysis to an average 3 patients per 24 hours with a very significant cost benefit to the health system.

Dr Gareth Davies, medical director of London HEMS, gave the thought provoking keynote address – *the air ambulance of the future*. The concept of ‘critical care without walls’ was again developed during this presentation. The idiosyncratic introduction of interventions into pre-hospital trauma practice was explored as well as the potential deviations from good in-hospital medical practice that this has produced. Overall developments of aeromedical systems have to reflect the effective delivery of time critical interventions when the patient needs them rather than when the EMS can comfortably provide them.

## Safety

The first afternoon session addressed patient safety in air ambulances. The delegates heard two talks from Dr Stephen Sollid, Dean of the Norwegian air ambulance academy, on current safety records and maintenance and improvement of standards in patient safety. The reduction of ‘human factor’ incidents’ with human factor training and the use of techniques from industry were discussed. The search for useful techniques that allow the benefits of effective emergency medicine in an unpredictable environment while delivering maximum patient and provider safety is a major challenge in air ambulance practice.

## Clinical

Professor Wolfgang Voelckel from Austria, spoke about pre-hospital interventions in traumatic brain injury, aimed at treating early coagulopathy as well as the traditional correction of hypoxaemia, hypercarbia and hypotension. Stating *best practice needs a physician*, Professor Voelckel argued that initial assessment and subsequent decision-making requires high levels of experience. The relationship between GCS and mortality is not linear; but the relationship between brain injury survival rate and decreased much more reliable. Delegates were invited to consider why we still use the full GCS score.

Advanced circulatory support in air ambulance transfer was examined in a presentation by Dr Mathias Zuercher, from Switzerland. He conducted a detailed examination of the devices for cardiac support and oxygenation that can be used for patient transfer between hospitals. Variability in levels of support, weight and battery life of devices were outlined in addition to the complexity of patients, and high risk of using extracorporeal cardiopulmonary devices with no back up.

The day concluded with a talk by Dr Anne Weaver, Clinical Lead at London’s Air Ambulance, on pre-hospital treatment of haemorrhage – what can a physician manned air ambulance bring to scene? She established that early identification and treatment of haemorrhage is possible using simple physiological criteria. Having identified patients with severe haemorrhage there is consensus that early administration of blood and blood products is preferable to crystalloid administration. A working system to provide blood on board a helicopter trauma service was described. Regular transfusions have been given and close co-operation with the major trauma centre haematological service have led to the highest standards of blood traceability and transfusion safety in the pre-hospital phase of care.

The content of the Air Ambulance day, which was a new event at the London Trauma Conference, brought together more than 200 delegates and speakers who aspire to the delivery of high quality care in the aeromedical environment. Although most had similar aims, the services represented approach the key areas of organisation and safety in different ways. The advance of techniques and technology mean that most in-hospital techniques can be conducted outside hospital. Establishing which interventions should be carried out will be a major challenge and is likely to be the subject of future similar days of discussion.

